# High seroprevalence of severe acute respiratory syndrome coronavirus 2 among healthcare workers in Yaoundé, Cameroon after the first wave of Covid‐19 pandemic and associated factors

**DOI:** 10.1111/irv.13239

**Published:** 2024-02-11

**Authors:** Mathurin Cyrille Tejiokem, Hermine Abessolo Abessolo, Joseph Mendimi Nkodo, Mireille Ouethy, Georges Bouting Mayaka, Yannick Touha, Ulrich Armel Dikoume, Jules Brice Tchatchueng‐Mbougua, Diane Choualeu Noumbissi, William Tsobeng Ndjeukam, Hervé Odilon Otabela Mbarga, Paul Alain Tagnouokam Ngoupo, Charlotte Moussi, Bonaventure Hollong Garoua, Richard Njouom, Vincent Richard

**Affiliations:** ^1^ Service d'épidémiologie et de santé publique Centre Pasteur du Cameroun Yaoundé Cameroon; ^2^ Centre Spécialisé de Prise en Charge des Patients Covid‐19 annexe 2 Hôpital Central de Yaoundé Yaoundé Cameroon; ^3^ Hôpital Jamot de Yaoundé Yaoundé Cameroon; ^4^ Hôpital de District d'Obala Obala Cameroon; ^5^ Hôpital de District de Mbalmayo Mbalmayo Cameroon; ^6^ Service de virologie Centre Pasteur du Cameroun Yaoundé Cameroon; ^7^ Délégation Régionale de la Santé Publique du Centre Yaoundé Cameroon; ^8^ Direction Internationale, Institut Pasteur, Réseau International des Instituts Pasteur Paris France

**Keywords:** antibody persistence, Cameroon, health personnel, SARS‐CoV‐2 infection antibody testing, seroepidemiologic studies

## Abstract

**Background:**

Healthcare workers (HWs) are at a high risk of exposure to emerging health threats. Following the first wave of the coronavirus disease 2019 pandemic in Cameroon, we explored the presence and persistence of naturally acquired antibodies against severe acute respiratory syndrome coronavirus 2 (SARS‐CoV‐2) and the factors associated with seropositivity in HWs.

**Methods:**

Staff at two referral hospitals in Yaoundé or two Health District Hospitals in Obala and Mbalmayo were included in a 6‐month prospective cohort analysis or cross‐sectional survey, respectively. Seroprevalence and associated factors were determined, and Kaplan–Meier curves and Cox proportional hazards models were used to assess antibody persistence or positive seroconversion over time.

**Results:**

From August 2020 to March 2021, 426 HWs (median age: 31 years, interquartile range: 27–37 years; 66.4% female) were enrolled. The overall seroprevalence of anti‐SARS‐CoV‐2 antibodies was 54.0% (95% confidence interval [CI]: 49.1–58.8) and was significantly different between study sites (*p* = 0.04). Of the 216 HWs included in the 6‐month cohort, 109 (50.5%) HWs were seropositive at inclusion; the probability of persistent antibodies or of becoming seropositive was 93.8% (95% CI: 84.2–100) and 78.9% (95% CI: 61.7–88.4), respectively. Seroconversion was associated with study site and occupation but not with infection prevention and control (IPC) practices.

**Conclusions:**

We observed high seroprevalence of SARS‐CoV‐2 antibody and seroconversion among HWs associated with occupational risk. This suggests low compliance to the COVID‐19 control measures. Continued training and implementation of IPC measures and accelerated preparedness are needed to better tackle future threats.

## 1 INTRODUCTION

Severe acute respiratory syndrome coronavirus 2 (SARS‐CoV‐2) is an ongoing threat to public health and healthcare systems.^1^ Having rapidly spread worldwide, the virus has affected more than 541 million people and caused over 6.3 million deaths at the time of writing.^2^ During the first wave of the coronavirus disease 2019 (COVID‐19) pandemic, healthcare workers (HWs) were at a higher risk of SARS‐CoV‐2 infection than the general population because of direct and indirect contact with patients, insufficient preparedness for managing a novel viral agent, a large workload with rapid changes in routine, and poor access to personal protective equipment (PPE).^3^

HWs were particularly affected during the early waves of the pandemic, with numbers likely underestimated because of undiagnosed asymptomatic infections.^4^ In Italy, 12% of the 250 495 COVID‐19 cases recorded up to August 10, 2020, were in HWs.^5^ In Cameroon, the first COVID‐19 case was identified on March 5, 2020, and by September 9, 2020, HWs accounted for 4% of the 20 009 cases identified.^6^ The World Health Organization (WHO) estimated that approximately 14% of SARS‐CoV‐2 infections occur in HWs during the initial wave of the pandemic.^7^ Failing to control infection among HWs leads to a significant risk of transmission to patients, colleagues, and social contacts, and the collapse of the healthcare system.^8^ Understanding the actual extent of infection among HWs is a prerequisite to controlling infection.

Seroprevalence studies of anti‐SARS‐CoV‐2 antibodies, particularly those performed before COVID‐19 vaccination (which started in Cameroon on April 12, 2021), make it possible to (1) understand the spread of infection among HWs within healthcare facilities and determine those at risk,^9^ (2) assess the effectiveness of interventions to help public health officials plan for future healthcare needs, and (3) design occupational strategies against potential new outbreaks. Many such seroprevalence studies have been conducted, with substantially different results between and within countries (0.7%–70.4%). This variation is due to differences in sampling, healthcare activity, testing periods, antibody detection kit performance, and the infection prevention and control (IPC) measures applied.^10^, ^11^, ^12^, ^13^

Few seroprevalence studies have been conducted on HWs in health facilities in Cameroon. We aimed to determine the seroprevalence of naturally acquired anti‐SARS‐CoV‐2 antibodies and evaluate their durability in a cohort of HWs over a 6‐month period. We also explored the factors associated with anti‐SARS‐CoV‐2 seropositivity.

## 2 METHODS

### 2.1 Study settings

This study was conducted at two referral hospitals in Yaoundé and two health district hospitals in Obala and Mbalmayo in Cameroon (Figure S1). Jamot Hospital in Yaoundé (JHY), a referral center dedicated to infectious respiratory diseases, created a new section for the diagnosis and care of suspected COVID‐19 cases. The Specialized Center for the Care of COVID‐19 Patients (SCCCP) ‐ Annex 2 of the Central Hospital in Yaoundé was built during the first wave of the pandemic to treat COVID‐19 exclusively.

The two district hospitals, Obala and Mbalmayo, are referral institutions peripheral to Yaoundé that also dedicated specific areas to the diagnosis and care of patients with COVID‐19; however, they had as supposed more limited access to PPE and IPC measures than JHY or SCCCP.

### 2.2 Study period and design

This study was conducted between August 2020 (after the first wave of the pandemic in Cameroon) and August 2021 (Figure S2). A prospective cohort analysis was conducted on HWs enrolled at different time points at JHY (August 24, 2020, to October 8, 2020) and SCCCP (December 1, 2020, to February 14, 2021) and followed‐up at their workplaces every month as planned for 6 months. Additionally, a cross‐sectional analysis was conducted at Mbalmayo (February 25, 2021, to March 1, 2021) and Obala (March 8, 2021, to March 10, 2021) (Table S1a). Our study protocol was an adaptation of the WHO UNITY studies that prospectively assessed the extent of SARS‐CoV‐2 infection and infection risk factors among HWs.^14^

### 2.3 Study population

We included any health, administrative, and support staff whose names were on the personnel list provided to our research team by the hospital authorities and who consented to participate in the study.

### 2.4 Data collection, blood sampling, and serological analysis

A self‐administered questionnaire was completed at inclusion by all participants under the supervision of the Site investigator, verified, and entered into a database with the support of the Clinical Monitor using an Open Data Kit mask on a tablet (server at the Centre Pasteur du Cameroun [CPC]). The questionnaire comprised sociodemographic information, medical history, history of symptoms compatible with COVID‐19 or COVID‐19 polymerase chain reaction (PCR) results from early March 2020, and IPC practices. Then, symptoms were collected every month during follow‐up.

About 3–5 mL whole blood was drawn into a dry tube and transferred to the Virology Laboratory at the CPC at inclusion and every month for 6 months as planned. The sample was centrifuged and sera were collected and stored at −80°C. Serological analyses were conducted in batches at the end of the study using the WANTAI SARS‐CoV‐2 IgG ELISA (Quantitative) assay (Beijing Wantai Biological Pharmacy, Beijing, China) to detect total anti‐SARS‐CoV‐2 antibodies. A WANTAI SARS‐CoV‐2 Ab ELISA validation study indicated a sensitivity and specificity of 96.7% and 97.5%, respectively.^15^ The results were calculated as the ratio of the optical density of the sample to that of the cutoff, as proposed by the manufacturer. The test was considered negative if the ratio was ≤1.1. The results were provided to each participant after they received an explanation.

### 2.5 Ethical considerations

The study protocol was approved by the National Ethics Committee for Human Health Research (approval number 2020/05/1225/CE/CNERSH/SP) and administrative authorization was granted by the Ministry of Public Health in Cameroon. All participants provided written informed consent.

### 2.6 Statistical analyses

Categorical and continuous variables were described using proportions and medians with interquartile ranges (IQR), respectively. HWs characteristics and their IPC practices at baseline (all four study sites) were compared according to anti‐SARS‐CoV‐2 total antibody status (seropositive vs. seronegative), sex (female vs. male), level of hospital care (district vs. referral) and age (>30 vs. ≤30 years) using chi‐squared or Fisher's exact tests, or Mann–Whitney *U* tests, as appropriate. The overall seroprevalence was calculated using serological results from samples collected during the cross‐sectional survey and those collected from the cohort participants at inclusion and was adjusted for testing error using the following equation^16^:

$$\text{adjusted prevalence}=\frac{\text{crude prevalence}+\text{specificity}–1}{\text{sensitivity}+\text{specificity}–1}.$$

Monthly seroprevalence was estimated considering samples collected from newly included HWs and tested during the identified month throughout the follow‐up period.

Univariate and multivariate logistic regression analyses were conducted to identify factors associated with total anti‐SARS‐CoV‐2 seropositivity in HWs at enrollment. Factors with a *p*‐value ≤ 0.2 in the univariate analysis were included in the multivariate regression. The threshold for statistical significance was set at *p* < 0.05 (two‐sided).

The Kaplan–Meier method was used to estimate the probability of SARS‐CoV‐2 antibody persistence or positive seroconversion over time among HWs of referral hospitals in Yaoundé. Multivariate adjusted hazard ratios (aHR) and 95% confidence intervals (CI) were estimated using Cox proportional hazards models to assess whether the two referral hospitals HWs' characteristics were associated with seroconversion over time. Statistical analyses were conducted using R software (R Foundation for Statistical Computing, Vienna, Austria; RRID:SCR_001905).

## 3 RESULTS

### 3.1 Study population

In total, 426 staff, representing 75.4% of the total HWs at participating sites, were enrolled in the study (Table S1a). The median age of HWs was 31.0 (IQR: 27.0–37.0 years), and women accounted for 66.4% (*n* = 283). Among HWs, 47.7% were paramedics (nurses, assistants, and lab technicians), and 13.1% were administrative employees, 13.1% were surface technician/Hygienist, 10.6% were medical practitioners, 9.2% were trainees (medical students, nurses, and laboratory technicians), and 6.6% were patient reception and transport staff (Table 1). Overall, 24.4% (*n* = 104) had already undergone at least one diagnostic PCR test, and eight declared that they tested positive for SARS‐CoV‐2. Approximately 16% (*n* = 69) of the HWs declared at least one pre‐existing condition, including obesity (7.8%), cardiac disease (1.6%), asthma (1.9%), diabetes (1.2%), HIV infection (1.2%), and pregnancy (2.6%). Regarding IPC practices, 45 to 67% of HWs declared that they always complied with the recommendations for each of the various practices. HWs from referral hospitals had better IPC practices including the use when indicated of infection protection equipment than those from district hospitals (Table S2a). However, no difference was observed when IPC practices were compared according to SARS‐CoV‐2 serostatus (seropositive vs. seronegative), sex (female vs. male), and age (<30 vs. ≥30 years) of HWs (Table S2a–c).

**TABLE 1:** Sociodemographic characteristics and history of Covid‐19 testing among health care workers at inclusion in four hospitals in Yaoundé and periphery, August 2020 to August 2021, Cameroon.

Characteristics	Health care workers SARS‐CoV‐2 serology						*p*‐value
	Total (*N* = 426)		Seronegative (*N* = 196)		Seropositive (*N* = 230)		
Age (median, IQR)	31.0	[27.0–37.0]	31.0	[27.0–36.0]	31.5	[27.3–38.0]	
Age group (years)	n	(%)	n	(%)	n	(%)	0.55
Less than first quartile (<27)	119	(28.0)	61	(51.3)	58	(48.7)	
First quartile‐median (27–31)	102	(24.0)	45	(44.1)	57	(55.9)	
Median third quartile (31–37)	100	(23.5)	45	(45.0)	55	(55.0)	
More than third quartile (>37)	104	(24.5)	44	(42.3)	60	(57.7)	
Gender							
Female	283	(66.4)	133	(47.0)	150	(53.0)	0.64
Male	143	(33.6)	63	(44.1)	80	(55.9)	
Occupied position							
Medical practitioner	45	(10.6)	32	(71.1)	13	(28.9)	0.004
Administrative personnel	56	(13.2)	27	(48.2)	29	(51.8)	
Lab technician	56	(13.2)	29	(51.8)	27	(48.2)	
Nurses and similar staff	93	(21.8)	39	(41.9)	54	(58.1)	
Care assistant	54	(12.7)	17	(31.5)	37	(68.5)	
Surface technician/hygienist	55	(12.9)	26	(47.3)	29	(52.7)	
Reception/transport	28	(6.6)	8	(28.6)	19	(71.4)	
Interns	39	(9.2)	18	(46.2)	21	(53.8)	
Comorbidities							
Obesity	33	(7.7)	22	(66. 7)	11	(33.3)	0.02
Cancer	0	(0.0)	0	(0.0)	0	(0.0)	/
Diabetes	5	(1.2)	4	(80.0)	1	(20.0)	0.23
HIV	4	(0.9)	2	(50.0)	2	(50.0)	0.35
Cardiac disease	7	(1.6)	4	(57.1)	3	(42.9)	0.09
Asthma	8	(1.9)	6	(75.0)	2	(25.0)	0.24
Chronic lung diseases	1	(0.2)	1	(100.0)	0	(0.0)	0.36
Chronic liver diseases	1	(0.2)	0	(0.0)	1	(0.0)	0.05
Chronic hematologic diseases	2	(0.5)	2	(100.0)	0	(0.0)	0.26
Pregnancy	11	(2.6)	4	(36.4)	7	(63.6)	0.70
Chronic kidney disease	1	(0.2)	0	(0.0)	1	(100.0)	0.65
Neurological deficiency	7	(1.6)	2	28.6%	5	(71.4)	0.27
Organ recipient	0	(0.0)	0	(0.0)	0	(0.0)	/
Other diseases	3	(0.7)	2	(66.7)	1	(33.3)	0.24
No comorbidity	357	(83.8)	156	(43.7)	201	(56.3)	0.04
One comorbidity	57	(13.4)	31	(54.4)	26	(45.6)	
More than one	12	(2.8)	9	(75.0)	3	(25.0)	
Study sites							0.04
District Hospital Obala	97	(22.8)	34	(35.1)	63	(64.9)	
District Hospital Mbalmayo	113	(26.5)	55	(48.7)	58	(51.3)	
Jamot Hospital Yaounde	61	(14.3)	35	(57.4)	26	(42.6)	
SCCCP de Yaoundé	155	(36.4)	72	(46.4)	83	(53.6)	

Abbreviation: SCCCP, Specialized Center for the Care of COVID‐19 Patients, Annex 2 Yaounde Central Hospital.

### 3.2 Seroprevalence and total anti‐SARS‐CoV‐2 antibody changes over time

Of the 426 HWs tested at enrollment, 230 were positive for anti‐SARS‐CoV‐2 antibodies (54.0%; 95% CI: 49.1–58.8). Seroprevalence was significantly different between study sites (*p* = 0.04) (Table 2) but not between referral hospitals in Yaoundé (50.5%) and peripheral Health District Hospitals (57.6%; *p* = 0.14). The monthly seroprevalence varied from 33.3 to 78.3% in the cohort analysis (Table S1b). All eight HWs with positive PCR test results before enrollment were seropositive; further, the median time between PCR and serological tests was 179 days (IQR: 116–179.5 days; range: 96–224 days). Seroprevalence was similar between HWs who had and had not been PCR‐tested before our study.

**TABLE 2:** Relation between the presence of anti‐SARS‐CoV‐2 total antibodies, and sociodemographic characteristics and coexisting disease of healthcare workers from two referral hospitals in Yaoundé (univariate and multivariate Cox regression), August 2020 to August 2021, Cameroon.

Characteristics	Presence of total antibodies anti‐SARS‐CoV‐2 among healthcare workers							
	cHR	95% CI	*p*‐value	aHR initial	95% CI	aHR final	95% CI	*p*‐value
Female	0.87	0.64–1.19	0.39					
Age > 30	0.98	0.73–1.33	0.92					
More than one comorbidity	0.73	0.48–1.10	0.13	0.82	0.50–1.36			
Study sites			<0.001					<0.001
Jamot Hospital Yaounde (ref.)	1							
SCCCP	2.14	1.48–3.10		2.23	1.42–3.52	2.54	1.69–3.82	
Infection prevention and control practices (Always as recommended/Most of the time, occasionally/rarely [ref])								
Are you following recommended hand hygiene practices?	0.92	0.68–1.25	0.59					
Do you use an alcohol‐based hand cleaner for hand hygiene?	1.03	0.76–1.39	0.84					
Do you use soap and water for hand hygiene?	0.92	0.68–1.25	0.59					
Do you use hand sanitizer or soap and water before touching a patient?	1.29	0.94–1.77	0.11	1.08	0.69–1.70			
Do you use hand sanitizer or soap and water before cleaning or aseptic operation?	1.34	0.97–1.84	0.07	1.07	0.67–1.71			
Do you use an alcohol‐based hand rub or soap and water after (risky) exposure to body fluids?	1.08	0.77–1.51	0.65					
Do you use an alcohol‐based hand product or soap and water after touching a patient?	1.08	0.76–1.54	0.65					
Do you use alcohol‐based hand sanitizer or soap and water after touching a patient's environment?	1.21	0.85–1.71	0.28					
Do you follow standard infection prevention and control precautions when in contact with a patient?	1.27	0.92–1.75	0.14	0.79	0.53–1.18			
Do you wear Infection Protection Equipment when indicated?	1.85	1.17–2.92	0.009	1.53	0.83–2.82			
Occupied position			0.1					0.044
Medical practitioner (ref.)	1			1		1		
Administrative/other	1.96	1.03–3.72		1.71	0.80–3.64	1.86	0.98–3.53	
Lab technician	1.61	0.91–2.84		2.73	1.46–5.09	2.40	1.32–4.34	
Nurses	1.82	1.07–3.12		1.80	1.03–3.14	1.79	1.04–3.07	
Nurse assistant	2.25	1.13–4.49		2.72	1.32–5.59	2.62	1.31–5.25	
Surface technician/hygienist	1.88	1.11–3.20		1.82	1.04–3.18	1.77	1.04–3.01	
Reception/transport	2.00	1.05–3.83		2.30	1.17–4.52	2.31	1.21–4.42	

Abbreviations: aHR, adjusted hazard ratio; cHR, crude Hazard ratio; CI, confidence interval; SCCCP, Specialized Center for the Care of COVID‐19 Patients, Annex 2 Yaounde Central Hospital.

**FIGURE 1:**
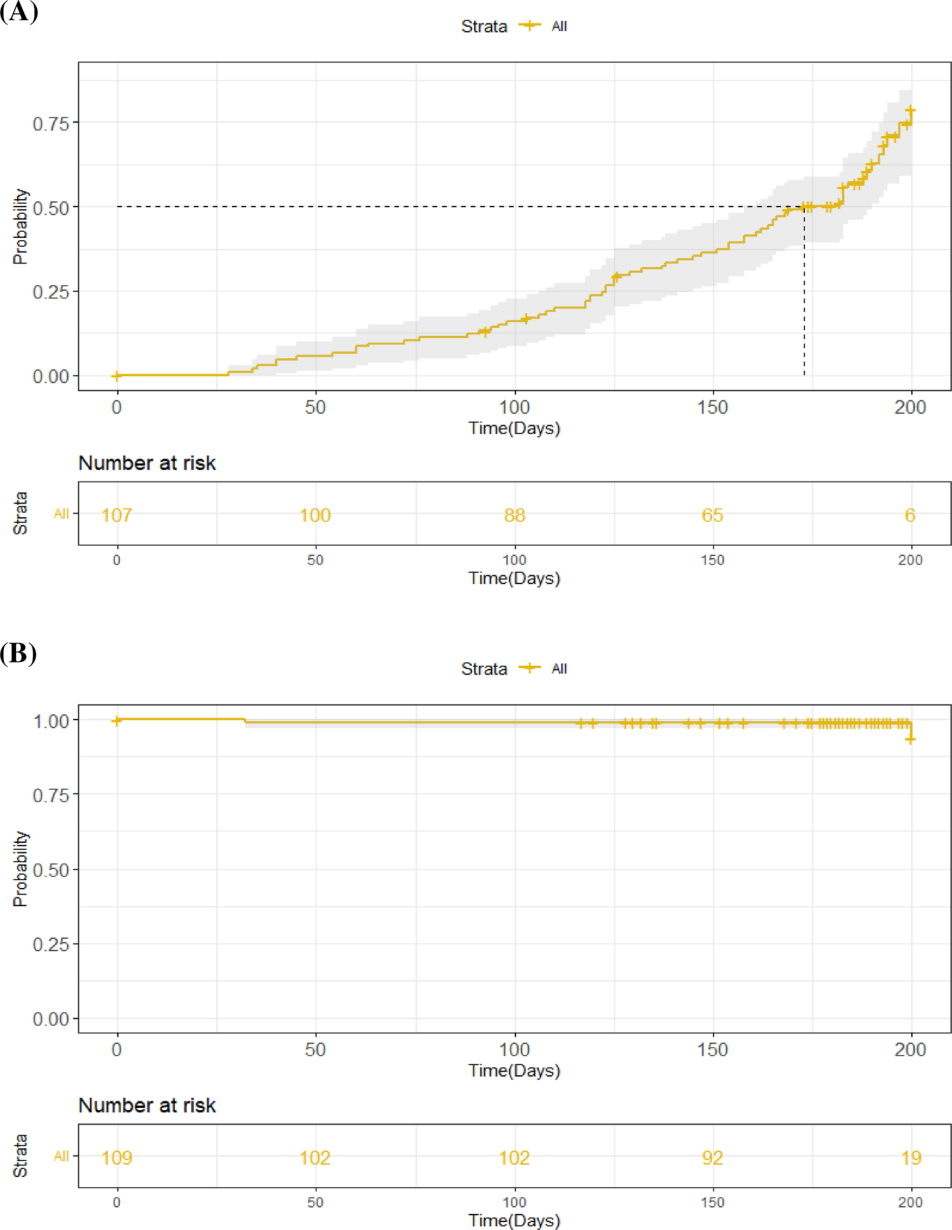
Kaplan Meier curves presenting (A) the probability of health care workers initially seronegative to become seropositive (B) the probability of health care workers initially seropositive to maintain their serological status over 6 months follow‐up, August 2020 to August 2021, Cameroon. The solid line indicates the probability and the shaded area represents the 95% confidence bands.

Of the 216 HWs from the referral hospitals in Yaoundé and included in the 6‐month cohort, 109 were seropositive at baseline. Six of these staff did not continue follow‐up, one became seronegative during follow‐up, and 102 remained seropositive throughout their follow‐up time in the study. The probability of persistent anti‐SARS‐CoV‐2 antibodies was 93.8% (95% CI: 84.2–100), as shown in the Kaplan–Meier curves (Figure 1). Of the 107 seronegative HWs, 66 seroconverted to seropositive status during follow‐up, resulting in an incidence of approximately 23 per 100 person‐months. Forty HWs remained negative throughout their follow‐up time and one was not followed. The probability of becoming seropositive over time was 78.9% (95% CI: 61.7–88.4; Figure 1). Among the 66 HWs who seroconverted during follow‐up, only 17 had symptoms that led to COVID‐19 diagnosis using SARS‐CoV‐2 PCR; the remaining 49 (74.2%) patients were asymptomatic. The median time from the first negative to the first positive serological test was 134.5 days (IQR: 98–166 days; range: 28–200 days).

### 3.3 Factors associated with seropositivity at baseline or seroconversion over time

Logistic regression (HWs from four sites involved at baseline) revealed that the absence of a pre‐existing condition (adjusted odds ratio [aOR]: 2.10; 95% CI: 1.18–3.73; *p* = 0.01), study site, and occupation were independently associated with seropositivity at enrollment. HWs from Obala had a higher risk of seropositivity (aOR: 2.01; 95% CI: 1.12–3.66; *p* = 0.02) than those from JHY. Additionally, compared with medical practitioners, lab technicians (aOR: 2.41; 95% CI: 1.02–5.74), nurses (aOR: 3.27; 95% CI: 1.49–7.15), nurse assistants (aOR: 5.16; 95% CI: 2.11–12.63), surface technicians/hygienists (aOR: 2.64; 95% CI: 1.12–6.18), and patient reception and transport staff (aOR: 5.92; 95% CI: 2.04–17.14) more frequently presented anti‐SARS‐CoV‐2 antibodies at baseline. No IPC practices were significantly associated with seropositivity at enrollment.

Table 2 presents the factors associated with seroconversion over time using Cox regression (HWs from two sites involved). The SCCCP study site was significantly associated with seroconversion (aHR: 2.54; 95% CI: 1.69–3.82; *p* < 0.001). Furthermore, compared with medical practitioners, nurses (aHR: 1.79; 95% CI: 1.04–3.07), nurse assistants (aHR: 2.62; 95% CI: 1.31–5.25), lab technicians (aHR: 2.54; 95% CI: 1.69–3.82; *p* < 0.001), patient reception and transport staff (aHR: 2.31; 95% CI: 1.21–4.42; *p* < 0.001), and surface technicians/hygienists (aHR: 1.77; 95% CI: 1.04–3.01) were at high risk for becoming seropositive over time. Finally, IPC practices were not associated with seroconversion.

## 4 DISCUSSION

The overall seroprevalence was 54%, ranging from 43 to 65% across the hospitals in our study. Comparable observations have been reported during similar periods elsewhere in Africa, particularly in Nigeria (45.1% seroprevalence),^17^ the Democratic Republic of Congo (41.2%),^18^ Ethiopia (39.6%),^19^ and South America (33.6%).^20^ However, our finding contrasts with the 17.9% seroprevalence observed in a cross‐sectional study of 368 HWs from three health district hospitals in Yaoundé conducted in January/February 2021.^21^ Moreover, low seroprevalence has been identified in most European and American countries.^11^, ^13^ This variation may be explained by differences in sampling techniques, healthcare activity, testing periods, antibody detection kits, and IPC measures.

No differences in seroprevalence between HWs working at referral sites in Yaoundé and those at peripheral health district hospitals (supposed to have limited access to PPE) were identified. This finding could be explained by the inappropriate use of PPE by referral hospital staff. A study conducted in China during the epidemic showed that hand hygiene and the appropriate use of masks, gloves, and gowns effectively prevented viral transmission in clinical settings.^22^ Furthermore, the seroprevalence observed in our analyses may be related to community transmission of SARS‐CoV‐2 through the household, social interactions, or shared office spaces. A sero‐survey of health district residents in Yaoundé conducted in October/November 2020, reported an adjusted anti‐SARS‐CoV‐2 IgG antibody seroprevalence of 29.2% (*n* = 971).^23^ Additionally, a population‐based survey conducted in Yaoundé in January/February 2021, reported a seroprevalence of 18.3% (150/722 participants from 393 households),^24^ indicating community transmission. However, this community seroprevalence was lower than that observed our study, which may reflect the high risk associated with hospital activities.

Anti‐SARS‐CoV‐2 antibody positivity at baseline was associated with health facility occupation, with a higher risk among nurses, assistant nurses, lab technicians, and patient reception/transport staff, indicating an occupational health risk among those in closest contact with patients. This could be explained by the longer duration of direct or indirect patient care, sometimes without appropriate protection. Similar observations regarding occupation were made in other studies, particularly in Egypt, where nurses and non‐clinical HWs were at a higher risk of seropositivity than doctors.^11^, ^25^ These findings demonstrate that all staff should be included in IPC training, regardless of their level of patient contact. No significant associations were observed between IPC practices and baseline seropositivity in our study. This lack of association, observed in other settings,^18^, ^26^ could have arisen from the subjective nature of our self‐administered questionnaire. In future studies, a combination of questionnaires and observation could help measure the true impact of IPC practices on infection transmission. This could explain the association between HW seropositivity and study site, as SCCCP had good access to PPE and registered a high seroconversion rate during follow‐up.

Most participants who had antibodies at enrollment or seroconverted during the study (74.2%) reported no prior symptoms and remained positive at the end of their follow‐up period. This large proportion of asymptomatic infections might have contributed to the nosocomial spread of the infection, indicating that the control of SARS‐CoV‐2 transmission requires the expansion of universal regular testing, particularly if strict adherence to preventive measures and continuous PPE training cannot be guaranteed.^27^

Almost all 109 HWs seropositive for anti‐SARS‐CoV‐2 antibodies at enrollment remained seropositive after an average follow‐up of 6 months. While this evidence is encouraging, whether these antibody levels confer protection against future SARS‐CoV‐2 infections is unclear, particularly considering emerging variants.^27^, ^28^, ^29^

After the first wave of the pandemic, more than half of the HWs in our study had anti‐SARS‐CoV‐2 antibodies. No clarity exists regarding what proportion of a population should be infected and/or vaccinated at a given time to achieve herd immunity.^30^, ^31^ Nevertheless, we hypothesize that the high positivity rate in our study could limit transmission among HWs. On the other hand, given the high seroprevalence, the consequences for the health system might have been extreme, had the fatality rate been as high as that for a more severe viral disease. Therefore, increased preparedness is required to better tackle future public health emergencies.

A major strength of this study is that more than three‐quarters of the total HWs at each site was included in our analysis, thus reducing potential population sampling bias. However, this study had some limitations. The time between SARS‐CoV‐2 exposure and initial antibody testing was unknown, which might have led to an underestimation of seroprevalence due antibodies waning. Additionally, we used commercial assays to detect total SARS‐CoV‐2 antibodies and were unable to determine whether the presence of these antibodies conferred protective herd immunity.^32^

In conclusion, our study showed a high seroprevalence, persistence of anti‐SARS‐CoV‐2 antibodies, and seroconversion among HWs after the first wave of the pandemic in Cameroon, which was associated with occupational risk, suggesting low compliance to the COVID‐19 control measures. Continued training and application of IPC measures in routine activities is essential for improved preparedness when facing future health threats.

## AUTHOR CONTRIBUTIONS

**Mathurin Cyrille Tejiokem:** Conceptualization; data curation; formal analysis; funding acquisition; methodology; project administration; software; validation; writing—original draft. **Hermine Abessolo Abessolo:** Data curation; investigation; writing—review and editing. **Joseph Mendimi Nkodo:** Investigation; supervision; writing—review and editing. **Mireille Ouethy:** Data curation; investigation; writing—review and editing. **Georges Bouting Mayaka:** Investigation; supervision; writing—review and editing. **Yannick Touha:** Data curation; investigation; writing—review and editing. **Ulrich Armel Dikoume:** Investigation; supervision; writing—review and editing. **Jules Brice Tchatchueng‐Mbougua:** Data curation; formal analysis; methodology; software; writing—review and editing. **Diane Choualeu Noumbissi:** Data curation; investigation; resources; writing—review and editing. **William Tsobeng Ndjeukam:** Data curation; investigation; resources; writing—review and editing. **Hervé Odilon Otabela Mbarga:** Data curation; investigation; resources; writing—review and editing. **Paul Alain Tagnouokam Ngoupo:** Resources; validation; writing—review and editing. **Charlotte Moussi:** Supervision; writing—review and editing. **Bonaventure Hollong Garoua:** Investigation; supervision; writing—review and editing. **Richard NJOUOM:** Supervision; writing—review and editing. **Vincent Richard:** Conceptualization; formal analysis; funding acquisition; methodology; software; supervision; writing—review and editing.

## CONFLICT OF INTEREST STATEMENT

The authors have no relevant financial or non‐financial interests to disclose.

## ETHICS STATEMENT

This study was approved by the Cameroon National Ethics Committee for Human Health Research (ethical approval no. 2020/05/1225/CE/CNERSH/SP). Written informed consent was obtained prior to the interview and sample collection. Patient consent statement for publication is not applicable. All records from this project were fully anonymized before researchers accessed them. This article is licensed under a Creative Commons Attribution 4.0 International License (http://creativecommons.org/licenses/by/4.0/), which permits use, sharing, adaptation, distribution and reproduction in any medium or format, as long as you give appropriate credit to the original author(s) and the source, provide a link to the Creative Commons license, and indicate if changes were made.

### PEER REVIEW

The peer review history for this article is available at https://www.webofscience.com/api/gateway/wos/peer-review/10.1111/irv.13239.

## Supporting information


**Figure S1.** a: Location of four hospitals involved in the healthcare workers study in Yaounde, Cameroon, August 2020 – August 2021.

**Figure S2:** Epidemic curve showing the case counts nationwide during the COVID‐19 pandemic. We mentioned on this curve different dates: the first COVID‐19 case detected, the first dose of COVID‐19 vaccination, launched of our study, and the vaccination coverage among HWs in Cameroon at the end of our study.

**Table S1.** a. Seroprevalence of anti‐SARS‐CoV‐2 antibodies among health care workers of four hospitals in Cameroon, August 2020 – August 2021.
**Table S1.** b. Monthly SARS‐CoV‐2 antibodies positivity rate among health care workers during study at four hospitals in Cameroon, August 2020 – August 2021.

**Table S2.** a. Infection prevention and control practices according to level of hospital care in Cameroon, August 2020–August 2021.
**Table S2.** b. Infection prevention and control practices at inclusion according to health workers gender at four hospitals in Cameroon, August 2020 – August 2021.
**Table S2.** c. Infection prevention and control practices at inclusion according to health workers age group at four hospitals in Cameroon, August 2020 – August 2021.
**Table S2.** d. Infection prevention and control practices according to health workers SARS‐CoV‐2 serostatus at four hospitals in Cameroon, August 2020 – August 2021.

## Data Availability

The data that support the findings of this study are available from the corresponding author upon request.
