# Supplementary figures and images for: High seroprevalence of severe acute respiratory syndrome coronavirus 2 among healthcare workers in Yaoundé, Cameroon after the first wave of Covid‐19 pandemic and associated factors
Source: Influenza Other Respir Viruses. 2024 Feb 11;18(2):e13239. doi: 10.1111/irv.13239 (PMC10859237; doi:10.1111/irv.13239)

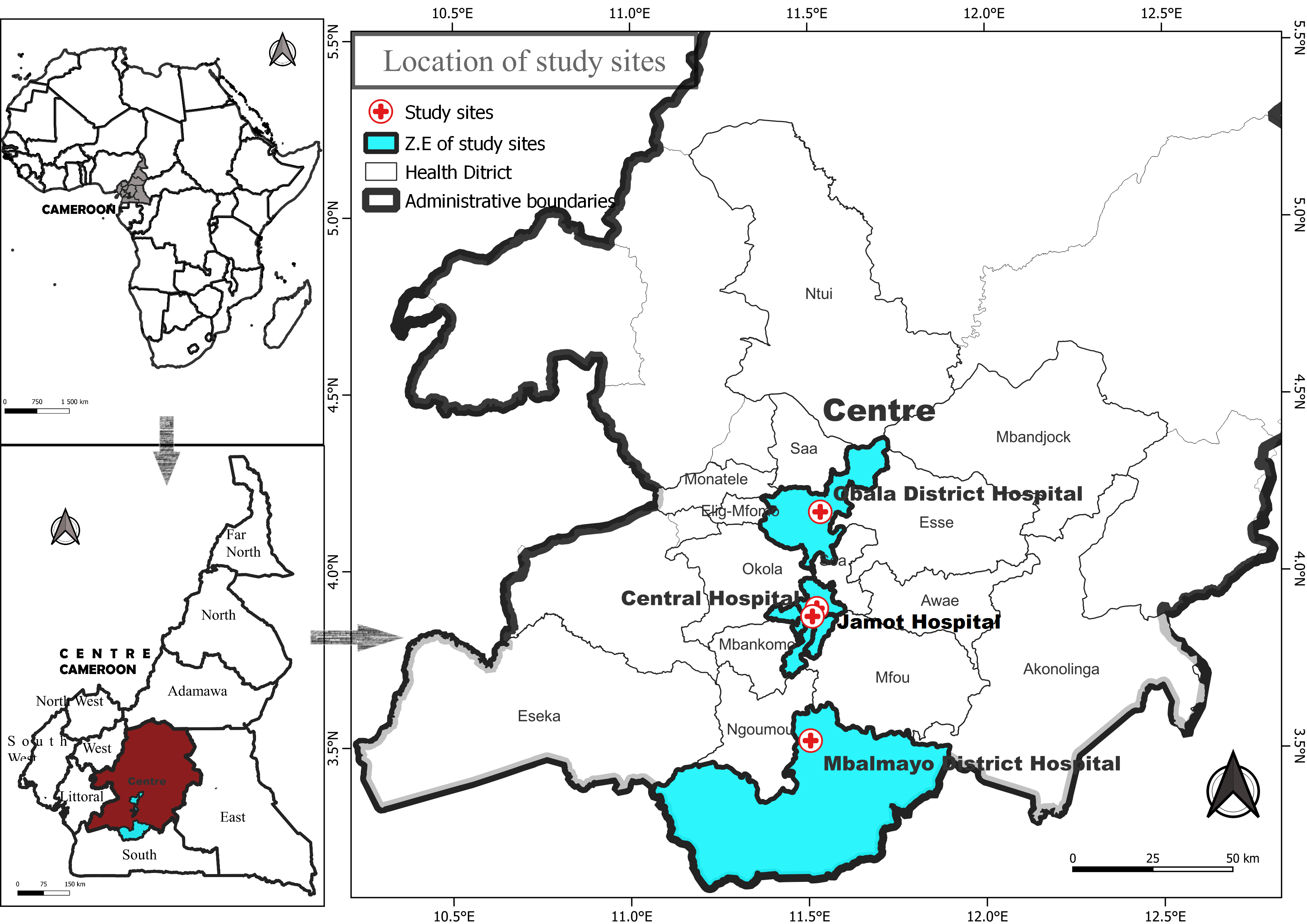

Supplement: Supplementary file 1 — Figure S1. a: Location of four hospitals involved in the healthcare workers study in Yaounde, Cameroon, August 2020 – August 2021. [file IRV-18-e13239-s001.png]

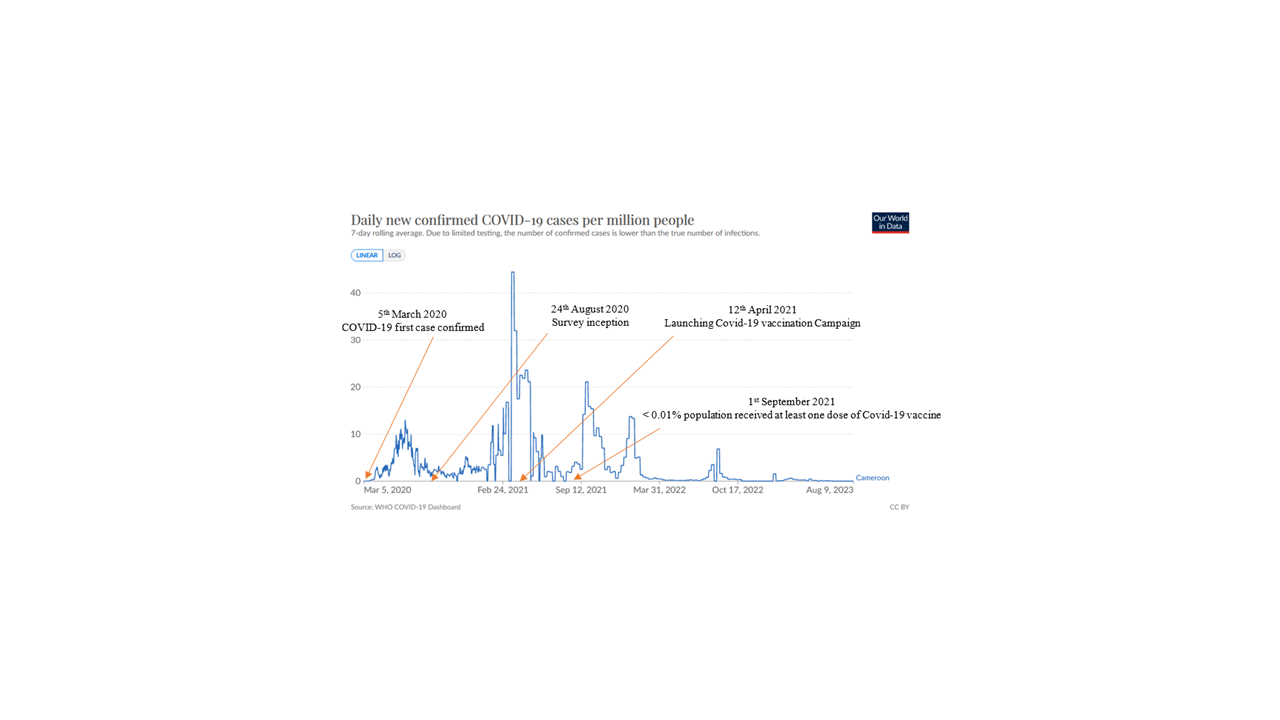

Supplement: Supplementary file 2 — Figure S2: Epidemic curve showing the case counts nationwide during the COVID‐19 pandemic. We mentioned on this curve different dates: the first COVID‐19 case detected, the first dose of COVID‐19 vaccination, launched of our study, and the vaccination coverage among HWs in Cameroon at the end of our study. [file IRV-18-e13239-s003.tif]
