# Supplementary material for: High seroprevalence of severe acute respiratory syndrome coronavirus 2 among healthcare workers in Yaoundé, Cameroon after the first wave of Covid‐19 pandemic and associated factors
Source: Influenza Other Respir Viruses. 2024 Feb 11;18(2):e13239. doi: 10.1111/irv.13239 (PMC10859237; doi:10.1111/irv.13239)
Supplement: Supplementary file 3 — Table S1. a. Seroprevalence of anti‐SARS‐CoV‐2 antibodies among health care workers of four hospitals in Cameroon, August 2020 – August 2021. Table S1. b. Monthly SARS‐CoV‐2 antibodies positivity rate among health care workers during study at four hospitals in Cameroon, August 2020 – August 2021. [file IRV-18-e13239-s004.docx]

**Table 1a.** Seroprevalence of anti-SARS-CoV-2 antibodies among health workers of four hospitals in Yaoundé and periphery, Cameroon, August 2020 – August 2021

| Sites |  | Total |  | Inclusion | | |  | Presence of anti-SARS-CoV-2 antibodies | Seroprevalence |
| --- | --- | --- | --- | --- | --- | --- | --- | --- | --- |
|  |  | N  565 |  | Start | End | Rate (%)  426 (75.4) |  | n | % (IC95%) |
| District Hospital Obala (DHO) |  | 135 |  | 08/03/2021 | 10/03/2020 | 97 (72) |  | 63 | 64.9 (54.6 - 74.4) |
| District Hospital Mbalmayo (DHM) |  | 175 |  | 25/02/2021 | 01/03/2021 | 113 (65) |  | 58 | 51.3 (41.7 - 60.8) |
| Jamot Hospital Yaounde (Covid-19 team) |  | 80 |  | 24/08/2020 | 08/10/2020 | 61 (76) |  | 26 | 42.6 (30.0 – 55.9) |
| SCCCP* de Yaoundé |  | 175 |  | 01/12/2020 | 14/02/2021 | 155 (89) |  | 83 | 53.6 (45.4 - 61.6) |

*SCCCP: *Specialized Center* for the *Care* of *COVID*-*19 Patients*, Annex 2 Yaoundé Central Hospital ; HDO and HDM are located at the periphery of Yaounde, less than 50 km from Yaoundé.

**Table 1b.** Monthly SARS-CoV-2 antibodies positivity rate among health workers during study at four hospitals in Yaoundé and periphery, Cameroon, August 2020 – August 2021

|  | **2020** | | | | |  | **2021** | | |  |  |
| --- | --- | --- | --- | --- | --- | --- | --- | --- | --- | --- | --- |
| Months | August | Sept | Oct | Nov | Dec |  | Jan | Feb | March |  | Total |
| Number Sampled | 15 | 41 | 5 | 0 | 132 |  | 23 | 85 | 125 |  | 426 |
| Number positive (%) | 5  (33.3%) | 18  (43.9%) | 3  (60.0 %) | 0 | 65  (49.2%) |  | 18  (78.3%) | 45  (52.9%) | 76  (49.2%) |  | 230  (54.0%) |
