# Supplementary material for: High seroprevalence of severe acute respiratory syndrome coronavirus 2 among healthcare workers in Yaoundé, Cameroon after the first wave of Covid‐19 pandemic and associated factors
Source: Influenza Other Respir Viruses. 2024 Feb 11;18(2):e13239. doi: 10.1111/irv.13239 (PMC10859237; doi:10.1111/irv.13239)
Supplement: Supplementary file 4 — Table S2. a. Infection prevention and control practices according to level of hospital care in Cameroon, August 2020–August 2021. Table S2. b. Infection prevention and control practices at inclusion according to health workers gender at four hospitals in Cameroon, August 2020 – August 2021. Table S2. c. Infection prevention and control practices at inclusion according to health workers age group at four hospitals in Cameroon, August 2020 – August 2021. Table S2. d. Infection prevention and control practices according to health workers SARS‐CoV‐2 serostatus at four hospitals in Cameroon, August 2020 – August 2021. [file IRV-18-e13239-s002.docx]

**Table 2a.** Infection prevention and control practices according to level of hospital care in Cameroon, August 2020-August 2021

|  |  |  | **Level of hospital care** | | | | | | |  |
| --- | --- | --- | --- | --- | --- | --- | --- | --- | --- | --- |
|  |  |  | **Total**  **(N=426)** | | **District**  **(n= 210)** | | **Referral**  **(n= 216)** | | **P-value** |  |
|  |  |  | **n** | **(%)** | **n** | **(%)** | **n** | **(%)** |  |  |
| **Are you following recommended hand hygiene practices?** | | |  |  |  |  |  |  |  |  |
|  | Always, as recommended | | 218 | (51.2) | 89 | (42.4) | 129 | (59.7) | <0.001 |  |
|  | Most of the time, occasionally, rarely | | 208 | (48.8) | 121 | (57.6) | 87 | (40.3) |  |  |
| **Do you use an alcohol-based hand cleaner for hand hygiene?** | | |  |  |  |  |  |  |  |  |
|  | Always, as recommended | | 212 | (49.8) | 96 | (45.7) | 116 | (53.7) | 0.10 |  |
|  | Most of the time, occasionally, rarely | | 214 | (50.2) | 114 | (54.3) | 100 | (46.3) |  |  |
| **Do you use soap and water for hand hygiene?** | | |  |  |  |  |  |  |  |  |
|  | Always, as recommended | | 240 | (56.3) | 108 | (51.4) | 132 | (61.1) | 0.04 |  |
|  | Most of the time, occasionally, rarely | | 186 | (43.7) | 102 | (48.6) | 84 | (38.9) |  |  |
| **Do you use hand sanitizer or soap and water before touching a patient? (n=416)** | | |  |  |  |  |  |  |  |  |
|  | Always, as recommended | | 212 | (51.0) | 91 | (43.5) | 121 | (58.4) | 0.02 |  |
|  | Most of the time, occasionally, rarely | | 204 | (49.0) | 118 | (56.5) | 86 | (41.6) |  |  |
| **Do you use hand sanitizer or soap and water before cleaning or aseptic operation? (n=415)** | | |  |  |  |  |  |  |  |  |
|  | Always, as recommended | | 223 | (53.7) | 97 | (46.4) | 126 | (61.2) | 0.003 |  |
|  | Most of the time, occasionally, rarely | | 192 | (46.3) | 112 | (53.6) | 80 | (38.8) |  |  |
| **Do you use an alcohol-based hand rub or soap and water after (risky) exposure to body fluids?(n=420)** | | |  |  |  |  |  |  |  |  |
|  | Always, as recommended | | 262 | (62.3) | 113 | (54.1) | 149 | (70.6) | <0.001 |  |
|  | Most of the time, occasionally, rarely | | 158 | (37.7) | 96 | (45.9) | 62 | (29.4) |  |  |
| **Do you use an alcohol-based hand product or soap and water after touching a patient? (n=415)** | | |  |  |  |  |  |  |  |  |
|  | Always, as recommended | | 261 | (62.9) | 112 | (53.6) | 149 | (72.3) | <0.001 |  |
|  | Most of the time, occasionally, rarely | | 154 | (37.1) | 97 | (46.4) | 57 | (27.7) |  |  |
| **Do you use alcohol-based hand sanitizer or soap and water after touching a patient's environment? (n=421)** | | |  |  |  |  |  |  |  |  |
|  | Always, as recommended | | 245 | (58.2) | 94 | (44.8) | 151 | (71.6) | <0.001 |  |
|  | Most of the time, occasionally, rarely | | 176 | (41.8) | 116 | (55.2) | 60 | (28.4) |  |  |
| **Do you follow standard infection prevention and control precautions when in contact with a patient? (n=419)** | | |  |  |  |  |  |  |  |  |
|  | Always, as recommended | | 187 | (44.6) | 54 | (25.7) | 133 | (63.6) | <0.001 |  |
|  | Most of the time, occasionally, rarely | | 232 | (55.4) | 156 | (74.3) | 76 | (36.4) |  |  |
| **Do you wear Infection Protection Equipment when indicated?** | | |  |  |  |  |  |  |  |  |
|  | Always, as recommended | | 285 | (66.9) | 105 | (50.0) | 180 | (83.3) | <0.001 |  |
|  | Most of the time, occasionally, rarely | | 141 | (33.1) | 105 | (50.0) | 36 | (16.7) |  |  |

**Table 2b.** Infection prevention and control practices at inclusion according to health workers gender at four hospitals in Cameroon, August 2020 – August 2021

|  | | | **Health workers gender** | | | | | | |  |
| --- | --- | --- | --- | --- | --- | --- | --- | --- | --- | --- |
|  | | | **Total**  **(N= 426)** | | **Female**  **(N=283)** | | **Male**  **(N=143)** | | **P-value** |  |
|  |  |  | **n** | **(%)** | **n** | **(%)** | **n** | **(%)** |  |  |
| **Are you following recommended hand hygiene practices?** | | |  |  |  |  |  |  |  |  |
|  | Always, as recommended | | 218 | (51.2) | 141 | (49.8) | 77 | (53.9) | 0.43 |  |
|  | Most of the time, occasionally, rarely | | 208 | (48.8) | 142 | (50.2) | 66 | (46.1) |  |  |
| **Do you use an alcohol-based hand cleaner for hand hygiene?** | | |  |  |  |  |  |  |  |  |
|  | Always, as recommended | | 212 | (49.8) | 139 | (49.1) | 73 | (51.1) | 0.70 |  |
|  | Most of the time, occasionally, rarely | | 214 | (50.2) | 144 | (50.9) | 70 | (48.9) |  |  |
| **Do you use soap and water for hand hygiene?** | | |  |  |  |  |  |  |  |  |
|  | Always, as recommended | | 240 | (56.3) | 158 | (55.8) | 82 | (57.3) | 0.77 |  |
|  | Most of the time, occasionally, rarely | | 186 | (43.7) | 125 | (44.2) | 61 | (42.7) |  |  |
| **Do you use hand sanitizer or soap and water before touching a patient?** | | |  |  |  |  |  |  |  |  |
|  | Always, as recommended | | 212 | (51.0) | 138 | (49.6) | 74 | (53.6) | 0.44 |  |
|  | Most of the time, occasionally, rarely | | 204 | (49.0) | 140 | (50.4) | 64 | (46.4) |  |  |
| **Do you use hand sanitizer or soap and water before cleaning or aseptic operation?** | | |  |  |  |  |  |  |  |  |
|  | Always, as recommended | | 223 | (53.9) | 151 | (54.5) | 72 | (52.2) | 0.65 |  |
|  | Most of the time, occasionally, rarely | | 192 | (46.1) | 126 | (45.5) | 66 | (47.8) |  |  |
| **Do you use an alcohol-based hand rub or soap and water after (risky) exposure to body fluids?** | | |  |  |  |  |  |  |  |  |
|  | Always, as recommended | | 262 | (62.2) | 189 | (67.5) | 73 | (52.1) | 0.002 |  |
|  | Most of the time, occasionally, rarely | | 158 | (37.8) | 91 | (32.5) | 67 | (47.9) |  |  |
| **Do you use alcohol-based hand sanitizer or soap and water after touching a patient?** | | |  |  |  |  |  |  | 0.70 |  |
|  | Always, as recommended | | 261 | (62.9) | 176 | (63.5) | 85 | (61.6) |  |  |
|  | Most of the time, occasionally, rarely | | 154 | (36.5) | 101 | (36.5) | 53 | (38.4) |  |  |
| **Do you use alcohol-based hand sanitizer or soap and water after touching a patient's environment?** | | |  |  |  |  |  |  |  |  |
|  | Always, as recommended | | 245 | (58.2) | 168 | (59.4) | 77 | (55.8) | 0.49 |  |
|  | Most of the time, occasionally, rarely | | 176 | (41.8) | 115 | (40.6) | 61 | (44.2) |  |  |
| **Do you follow standard infection prevention and control precautions when in contact with a patient?** | | |  |  |  |  |  |  |  |  |
|  | Always, as recommended | | 187 | (44.6) | 119 | (42.7) | 68 | (48.6) | 0.25 |  |
|  | Most of the time, occasionally, rarely | | 232 | (55.4) | 160 | (57.4) | 72 | (51.4) |  |  |
| **Do you wear Infection Protection Equipment when indicated?** | | |  |  |  |  |  |  |  |  |
|  | Always, as recommended | | 285 | (66.9) | 191 | (67.5) | 94 | (65.7) | 0.72 |  |
|  | Most of the time, occasionally, rarely | | 141 | (33.1) | 92 | (32.5) | 49 | (34.3) |  |  |

**Table 2c.** Infection prevention and control practices at inclusion according to health workers age group at four hospitals in Cameroon, August 2020-August 2021

|  |  |  | **Health workers age (years)** | | | | | | |  |
| --- | --- | --- | --- | --- | --- | --- | --- | --- | --- | --- |
|  |  |  | **Total**  **(N= 426)** | | **< 30**  **(n=174)** | | ≥ **30**  **(n=252)** | | **P-value** |  |
|  |  |  | **n** | **(%)** | **n** | **(%)** | **n** | **(%)** |  |  |
| **Are you following recommended hand hygiene practices?** | | |  |  |  |  |  |  |  |  |
|  | Always, as recommended | | 218 | (51.2) | 85 | (48.9) | 133 | (52.8) | 0.43 |  |
|  | Most of the time, occasionally, rarely | | 208 | (48.8) | 89 | (51.1) | 119 | (47.2) |  |  |
| **Do you use an alcohol-based hand cleaner for hand hygiene?** | | |  |  |  |  |  |  |  |  |
|  | Always, as recommended | | 212 | (49.8) | 87 | (50.0) | 125 | (49.6) | 0.94 |  |
|  | Most of the time, occasionally, rarely | | 214 | (50.2) | 87 | (50.0) | 127 | (50.4) |  |  |
| **Do you use soap and water for hand hygiene?** | | |  |  |  |  |  |  |  |  |
|  | Always, as recommended | | 240 | (56.3) | 94 | (54.0) | 146 | (57.9) | 0.42 |  |
|  | Most of the time, occasionally, rarely | | 186 | (43.7) | 80 | (46.0) | 106 | (42.1) |  |  |
| **Do you use hand sanitizer or soap and water before touching a patient?** | | |  |  |  |  |  |  |  |  |
|  | Always, as recommended | | 212 | (51.0) | 87 | (50.9) | 125 | (51.0) | 0.98 |  |
|  | Most of the time, occasionally, rarely | | 204 | (49.0) | 84 | (49.1) | 120 | (49.0) |  |  |
| **Do you use hand sanitizer or soap and water before cleaning or aseptic operation?** | | |  |  |  |  |  |  |  |  |
|  | Always, as recommended | | 223 | (53.7) | 93 | (54.4) | 130 | (53.3) | 0.82 |  |
|  | Most of the time, occasionally, rarely | | 192 | (46.3) | 78 | (45.6) | 114 | (46.7) |  |  |
| **Do you use an alcohol-based hand rub or soap and water after (risky) exposure to body fluids?** | | |  |  |  |  |  |  |  |  |
|  | Always, as recommended | | 262 | (62.4) | 107 | (62.2) | 155 | (62.5) | 0.95 |  |
|  | Most of the time, occasionally, rarely | | 158 | (37.6) | 65 | (37.8) | 93 | (37.5) |  |  |
| **Do you use an alcohol-based hand product or soap and water after touching a patient?** | | |  |  |  |  |  |  |  |  |
|  | Always, as recommended | | 261 | (62.9) | 104 | (61.2) | 157 | (64.1) | 0.55 |  |
|  | Most of the time, occasionally, rarely | | 154 | (37.1) | 66 | (38.8) | 88 | (35.9) |  |  |
| **Do you use alcohol-based hand sanitizer or soap and water after touching a patient's environment?** | | |  |  |  |  |  |  |  |  |
|  | Always, as recommended | | 245 | (58.2) | 99 | (57.2) | 146 | (58.9) | 0.74 |  |
|  | Most of the time, occasionally, rarely | | 176 | (41.8) | 74 | (42.8) | 102 | (41.1) |  |  |
| **Do you follow standard infection prevention and control precautions when in contact with a patient?** | | |  |  |  |  |  |  |  |  |
|  | Always, as recommended | | 187 | (44.6) | 83 | (48.3) | 104 | (42.1) | 0.21 |  |
|  | Most of the time, occasionally, rarely | | 232 | (55.4) | 89 | (51.7) | 143 | (57.9) |  |  |
| **Do you wear Infection Protection Equipment when indicated?** | | |  |  |  |  |  |  |  |  |
|  | Always, as recommended | | 285 | (66.9) | 119 | (68.4) | 166 | (65.9) | 0.59 |  |
|  | Most of the time, occasionally, rarely | | 141 | (33.1) | 55 | (31.6) | 86 | (34.1) |  |  |

**Table 2d.** Infection prevention and control practices according to health workers SARS-CoV-2 serostatus at four hospitals in Cameroon, August 2020-August 2021

|  |  |  | **Health workers anti-SARS-CoV-2 antibody serology** | | | | | | |  |
| --- | --- | --- | --- | --- | --- | --- | --- | --- | --- | --- |
|  |  |  | **Total**  **(N=426)** | | **Negative**  **(n= 196)** | | **Positive**  **(n= 230)** | | **P-value** |  |
|  |  |  | **n** | **(%)** | **n** | **(%)** | **n** | **(%)** |  |  |
| **Are you following recommended hand hygiene practices?** | | |  |  |  |  |  |  |  |  |
|  | Always, as recommended | | 218 | (51.2) | 109 | (55.6) | 109 | (47.4) | 0.09 |  |
|  | Most of the time, occasionally, rarely | | 208 | (48.8) | 87 | (44.4) | 121 | (52.6) |  |  |
| **Do you use an alcohol-based hand cleaner for hand hygiene?** | | |  |  |  |  |  |  |  |  |
|  | Always, as recommended | | 212 | (49.8) | 97 | (49.5) | 115 | (50.0) | 0.91 |  |
|  | Most of the time, occasionally, rarely | | 214 | (50.2) | 99 | (50.5) | 115 | (50.0) |  |  |
| **Do you use soap and water for hand hygiene?** | | |  |  |  |  |  |  |  |  |
|  | Always, as recommended | | 240 | (56.3) | 114 | (58.2) | 126 | (54.8) | 0.48 |  |
|  | Most of the time, occasionally, rarely | | 186 | (43.7) | 82 | (41.8) | 104 | (45.2) |  |  |
| **Do you use hand sanitizer or soap and water before touching a patient?** | | |  |  |  |  |  |  |  |  |
|  | Always, as recommended | | 212 | (51.0) | 94 | (49.2) | 118 | (52.4) | 0.51 |  |
|  | Most of the time, occasionally, rarely | | 204 | (49.0) | 97 | (50.8) | 107 | (47.6) |  |  |
| **Do you use hand sanitizer or soap and water before cleaning or aseptic operation?** | | |  |  |  |  |  |  |  |  |
|  | Always, as recommended | | 223 | (53.7) | 99 | (52.1) | 124 | (55.1) | 0.54 |  |
|  | Most of the time, occasionally, rarely | | 192 | (46.3) | 91 | (47.9) | 101 | (44.9) |  |  |
| **Do you use an alcohol-based hand rub or soap and water after (risky) exposure to body fluids?** | | |  |  |  |  |  |  |  |  |
|  | Always, as recommended | | 262 | (62.3) | 122 | (63.2) | 140 | (61.7) | 0.74 |  |
|  | Most of the time, occasionally, rarely | | 158 | (37.7) | 71 | (36.8) | 87 | (38.3) |  |  |
| **Do you use an alcohol-based hand product or soap and water after touching a patient?** | | |  |  |  |  |  |  |  |  |
|  | Always, as recommended | | 261 | (62.9) | 121 | (63.7) | 140 | (62.2) | 0.75 |  |
|  | Most of the time, occasionally, rarely | | 154 | (37.1) | 69 | (36.3) | 85 | (37.8) |  |  |
| **Do you use alcohol-based hand sanitizer or soap and water after touching a patient's environment?** | | |  |  |  |  |  |  |  |  |
|  | Always, as recommended | | 245 | (58.2) | 120 | (61.9) | 125 | (55.1) | 0.66 |  |
|  | Most of the time, occasionally, rarely | | 176 | (41.8) | 74 | (38.1) | 102 | (44.9) |  |  |
| **Do you follow standard infection prevention and control precautions when in contact with a patient?** | | |  |  |  |  |  |  |  |  |
|  | Always, as recommended | | 187 | (44.6) | 88 | (46.1) | 99 | (43.4) | 0.59 |  |
|  | Most of the time, occasionally, rarely | | 232 | (55.4) | 103 | (53.9) | 129 | (56.2) |  |  |
| **Do you wear Infection Protection Equipment when indicated?** | | |  |  |  |  |  |  |  |  |
|  | Always, as recommended | | 285 | (66.9) | 133 | (67.9) | 152 | (66.1) | 0.70 |  |
|  | Most of the time, occasionally, rarely | | 141 | (33.1) | 63 | (32.1) | 78 | (33.9) |  |  |
